# The influence of metabolic syndrome on age-related hearing loss from the perspective of mitochondrial dysfunction

**DOI:** 10.3389/fnagi.2022.930105

**Published:** 2022-07-29

**Authors:** Dongye Guo, Andi Zhang, Tianyuan Zou, Rui Ding, Kaili Chen, Yi Pan, Peilin Ji, Bin Ye, Mingliang Xiang

**Affiliations:** ^1^Department of Otolaryngology & Head and Neck Surgery, Ruijin Hospital, Shanghai Jiao Tong University School of Medicine, Shanghai, China; ^2^Ear Institute, Shanghai Jiao Tong University School of Medicine, Shanghai, China; ^3^Shanghai Key Laboratory of Translational Medicine on Ear and Nose Diseases, Shanghai, China

**Keywords:** age-related hearing loss, metabolic syndrome, mitochondrial dysfunction, cochlea, mitochondria

## Abstract

With the increase in life expectancy in the global population, aging societies have emerged in many countries, including China. As a common sensory defect in the elderly population, the prevalence of age-related hearing loss and its influence on society are increasing yearly. Metabolic syndrome is currently one of the main health problems in the world. Many studies have demonstrated that metabolic syndrome and its components are correlated with a variety of age-related diseases of the peripheral sensory system, including age-related hearing loss. Both age-related hearing loss and metabolic syndrome are high-prevalence chronic diseases, and many people suffer from both at the same time. In recent years, more and more studies have found that mitochondrial dysfunction occurs in both metabolic syndrome and age-related hearing loss. Therefore, to better understand the impact of metabolic syndrome on age-related hearing loss from the perspective of mitochondrial dysfunction, we reviewed the literature related to the relationship between age-related hearing loss and metabolic syndrome and their components to discern the possible role of mitochondria in both conditions.

## Introduction

Age-related hearing loss (ARHL), also called presbycusis, is sensorineural hearing loss that results from the degeneration of the peripheral and/or central auditory nervous systems. ARHL is typically bilateral, symmetrical, and most pronounced at higher frequencies. Among people aged > 65 years, approximately one-third suffer from varying degrees of hearing loss (He et al., 2019). According to the seventh population census of China (2020), people aged ≥ 60 years account for 18.7% of the total population, reaching 264 million, and the number of those aged ≥ 65 years has increased by 4.63% since the sixth population census^1^. This trend indicates that an aging society has emerged in China. As the most common chronic sensory deficit in elderly individuals, ARHL is related to many physical and mental health problems (Bowl and Dawson, 2019). ARHL can be affected by metabolic diseases, noise, ototoxic drugs, heavy metals, and many other factors (He et al., 2019). At present, there is no specific medicine for the treatment of ARHL. Metabolic syndrome (MetS) generally includes diabetes, dyslipidemia, obesity, and hypertension (Saklayen, 2018). Approximately one-third of adults in the United States suffer from MetS (Saklayen, 2018). MetS and its components are closely related to a variety of age-related diseases of the peripheral sensory system, such as ARHL, cataract, and retinopathy (Poh et al., 2016; Kim et al., 2017). Patients with MetS often present with more progressive ARHL (Kim et al., 2017). More and more studies have shown that the development of ARHL may be due to the reduction in mitochondrial function (Chen and Tang, 2014), and mitochondrial dysfunction may also be a vital event in MetS (Prasun, 2020). Therefore, we consider that the effect of MetS on ARHL may be related to mitochondrial dysfunction. However, few studies to date have explored the molecular mechanism of MetS in ARHL. This review sought to summarize the correlation between MetS and its components and ARHL from the perspective of mitochondrial dysfunction and reveal the mechanism of how MetS affect ARHL through mitochondrial dysfunction.

## Mitochondrial function changes in the cochleae of age-related hearing loss (ARHL) models

**FIGURE 1:**
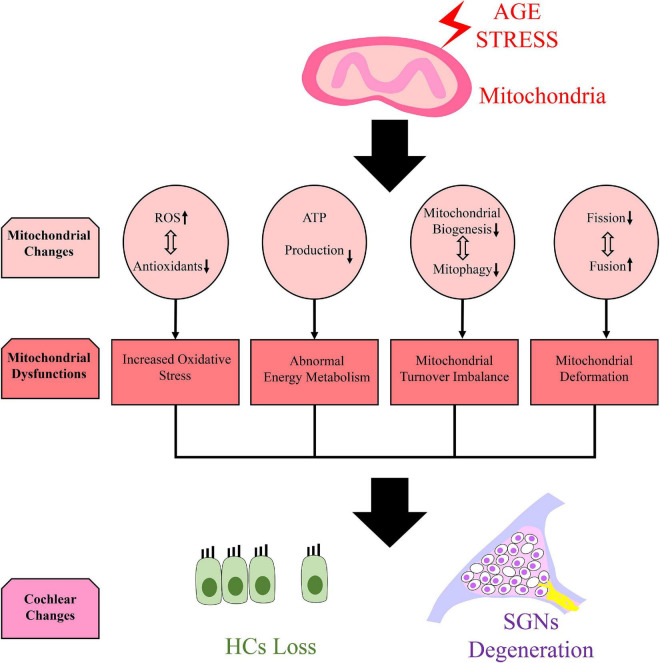
Mitochondrial dysfunction and cochlear changes caused by age stress. We speculate that mitochondrial dysfunction which manifests as increased oxidative stress (Chen et al., 2017), abnormal energy metabolism (Guo et al., 2020), imbalance mitochondrial turnover (Oh et al., 2020) and mitochondrial deformation (Lin et al., 2019) can be found in cochlea due to several mitochondrial changes caused by age stress. It can lead to hair cells (HCs) loss and spiral ganglion neurons (SGNs) degeneration in cochlea.

Age-related mitochondrial function changes may aggravate the development of age-related neurodegenerative diseases, and ARHL is directly related to mitochondrial dysfunction in cochlear cells (Chen and Tang, 2014). Mitochondrial dysfunction was found in ARHL models of cochlear explants, leading to the reduction of adenosine triphosphate (ATP) production (Guo et al., 2020). In aged auditory cortex neurons, oxygen species (ROS) production increased and the expression and activity of antioxidant enzymes decreased, showing an imbalance of oxidative stress homeostasis (Chen et al., 2017). In aged HEI-OC1 cell models induced by hydrogen peroxide, a disruption of the fission/fusion balance was caused by the reduction of mitochondrial fission–related protein expression, and mitochondria were swollen and pale (Lin et al., 2019). An imbalance in mitochondrial turnover was also found in aged cochleae because mitochondrial biogenesis and mitophagy were both inhibited (Oh et al., 2020) (Figure 1).

## Metabolic syndrome (MetS) is a risk factor for age-related hearing loss (ARHL)

Although there have been many articles that reviewed the studies about the relationship between MetS/its components and hearing loss now, but the number of articles can be greatly reduced when it comes to ARHL. We summarized 15 studies (eight human studies and seven animal studies, see Tables 1, 2) which designed and analyzed the ARHL and cochlear changes affected by MetS or its components. Through this table, it was found that MetS and its components can all have significant influence on ARHL, as mentioned below.

**TABLE 1:** Main human studies about the relationship between metabolic syndrome (MetS)/its components and age-related hearing loss (ARHL).

Study design	Number of subjects	Age (year)	Country	Associated components	Duration of follow-up	Main results
Cross-sectional study and longitudinal study (Mitchell et al., 2009)	1,923 subjects from the Blue Mountains Hearing Study	≥ 49	Australia	Diabetes	5 years	Cross-sectional results: Type 2 diabetes was independently associated with hearing loss (*P* < 0.05); the odds ratio for the association between diabetes and hearing loss for diabetes duration ≥ 10 years was higher than that for diabetes duration < 10 years. Longitudinal results: The progression of hearing impairment (>5dB hearing loss in pure-tone average at 0.5–4 KHz) was significant in subjects with newly diagnosed diabetes compared with those without diabetes (*P* = 0.04).
Cross-sectional study (Sommer et al., 2017)	1,864 participants from the Busselton Health Aging Survey	56.2 ± 5.5	Australia	Dysglycaemia	NA	Dysglycaemia independently impairs hearing at mid-range frequencies (500–4000 Hz) in subjects < 60 years with prediabetes or diabetes.
Cross-sectional study (Kim et al., 2020a)	16,779 subjects from the Korea National Health and Nutrition Examination Survey	>20	Korea	Diabetes/Central obesity/Hypertension	NA	The prevalence of hearing impairment was statistically related to the age (*P* < 0.0001). Hypertension, diabetes, and abdominal obesity, were significantly associated with hearing loss (*P* < 0.0001).
Cross-sectional study and longitudinal study (Simpson et al., 2013)	837 subjects for the cross-sectional study, 405 subjects for the longitudinal study	Mean age: 67.5	United States	Dyslipidemia (TC, HDL-C, triglyceride)	Every 2 or 3 years	Cross-sectional results: There was a positive association between TG and narrow PTA (*P* = 0.003), broad PTA (*P* < 0.001), low-frequency PTA (*P* = 0.002) and high-frequency PTA (*P* < 0.001). The TC/HDL-C ratio had a positive association with narrow PTA (P = 0.042), broad PTA (P = 0.03) and high-frequency PTA (P = 0.007). Longitudinal results: There were no significant associations between hearing and lipid levels.
Cross-sectional study and longitudinal study (Croll et al., 2019)	2,906 subjects for the cross-sectional study, 636 subjects for the longitudinal study	Mean age: 66.1	Netherlands	Obesity (BMI)	Median 4.4 years	Cross-sectional results: One-point higher BMI was associated with a 0.53 dB (CI: 0.04, 1.01) increase in hearing thresholds across all frequencies (0.25, 0.50, 1, 2, 4, and 8 kHz). Longitudinal results: BMI were not related to change in hearing thresholds at follow-up.
Cross-sectional study (Przewozny et al., 2016)	32 arterial hypertension patients and 32 age and sex-matched healthy volunteers	Arterial hypertension group: 53.1 ± 10.3, control group: 52.8 ± 11.4	Czech Republic	Hypertension	NA	Hearing thresholds of the hypertensive patients were higher than the control group (*P* < 0.05). DPOAE results of the hypertensive patients were significantly lower for frequencies 4 (P = 0.04) and 6 kHz (*P* < 0.001). The average sound localization ability was worse in the hypertensive patients and the differences were dependent on the symmetry and the severity of hearing loss.
Cross-sectional study (Rim et al., 2021)	94,223 subjects	Mean age of group with MetS: 46.1; mean age of group without MetS: 43.9	Korea	MetS/its components	NA	Subjects with MetS had higher mean pure-tone hearing thresholds than subjects without MetS in all age groups (*P* < 0.001). Among MetS subjects, there was a significant association between the number of MetS diagnostic components met and the hearing loss rate (*P* < 0.001). Among all diagnostic components, waist circumference, blood pressure, triglyceride and fasting blood sugar concentrations were strongly associated with hearing loss (*P* < 0.001).
Cross-sectional study (Han et al., 2018)	18,824 middle-aged and older subjects from the Dongfeng-Tongji Cohort study	Mean age: 64.7	China	MetS/its components	NA	Subjects with MetS had higher OR of hearing loss compared with subjects without MetS (*P* = 0.004). Among all diagnostic components, central obesity (*P* = 0.017), low HDL-C levels (*P* = 0.018), and hyperglycemia (*P* = 0.001) were all significantly associated with hearing loss.

PTA, pure-tone average; ABR, auditory brainstem response; DPOAE, distortion product otoacoustic emission; TC, total-cholesterol; LDL-C, LDL-cholesterol; HDL-C, HDL-cholesterol; TG, triglyceride; NA, not available.

**TABLE 2:** Main animal studies about the relationship between metabolic syndrome (MetS)/its components and hearing loss [mainly age-related hearing loss (ARHL)].

Experimental subjects	Testing items and time	Experimental design	Associated components	Main results-metabolic changes	Main results-hearing loss	Main results-cochlear changes
CBA/CaJ mice, male, 12 months old, *N* = 36 (Vasilyeva et al., 2009)	Blood glucose, body weight, ABR, DPOAE were measured at baseline, 2, 4 and 6 months (12, 14, 16, and 18 months of age).	The control group: *N* = 14, injected with citrate buffer and fed a standard Purina rodent chow and water *ad libitum* for 6 months; the T1DM group: *N* = 11, a single intraperitoneal injection of streptozotocin; the T2DM group: *N* = 11, fed a high fat, high simple carbohydrate, low fiber diet for 6 months	Diabetes (type 1 diabetes/type 2 diabetes)	Both the type 1 diabetes mellitus group (*P* < 0.001) and the type 2 diabetes mellitus group (*P* < 0.01) showed an elevation of fasting blood glucose by 6 months while the control group showed no elevation.	The ABR thresholds of the type 2 diabetes mellitus group elevated significantly (*P* < 0.05). Both the T1DM group (*P* < 0.001) and the T2DM group (*P* < 0.01) showed a significant drop in DPOAE at higher frequencies (23–45KHz) at 6 months compared to baseline.	NA
db/db mice, male, *N* = 22, db/ + mice, male, *N* = 20 (Lyu et al., 2021)	Measured ABR every week from 6 weeks of age, dissected the cochlea for histological and biological assays at 14 weeks of age.	The control group: db/ + mice, the diabetic group: db/db mice Both groups were fed pelleted food and water *ad libitum*.	Diabetes	The diabetic group showed increased body weight and hyperglycemia.	The ABR threshold was significantly increased in the diabetic group.	A significant higher loss of HCs in the basal turn, a reduction in the number of synaptic ribbons, a decreased cochlear blood flow and increased mitochondrial-mediated apoptosis were observed in db/db mice.
C57BL/6J mice and ApoE KO mice, male, 6 weeks old (Kim et al., 2020b)	Measured levels of plasma TC, HDL-C, LDL-C, TG at 8, 16, and 24 weeks of age; dissected the cochlea at 24 weeks of age.	Fed C57BL/6J mice continuously with a chow diet; group 1: *N* = 12, fed ApoE KO mice continuously with a chow diet; group2: *N* = 12, fed ApoE KO mice continuously with a western-type diet; all groups were fed for 16 weeks from 8 weeks of age	Dyslipidemia (TC, HDL-C, LDL-C, TG)	At 24 weeks of age, TC, LDL-C, HDL-C and TG were significantly increased in the group 2 compared to the control group.	At 24 weeks of age, the ABR thresholds in the left ear were significantly elevated in the group 2 compared with the control group and the group 1. The ABR thresholds were significantly correlated with TC (*P* < 0.001), LDL-C (*P* < 0.001), HDL- C (*P* < 0.001) and TG (*P* = 0.038) in all groups at 24 weeks of age.	It showed a significant increase of apoptosis in the SGNs of group 2.
Sprague–Dawley rats, male, 5 weeks old, *N* = 14 (Du et al., 2012)	Measured weight, plasma total cholesterol (TC) and triglycerides (TG) and dissected the cochlea at the end of the 12-month experimental period and 12 h after the last feeding; tested ABR both before and after the 12-month diet	The control group, the D-gal group, the HFD group and the D-gal + HFD group (*N* = 26 for each group) The control group and the D-gal group: fed a basic diet, the HFD group and the d-gal + HFD group: fed a high-fat diet for 12 months; t the D-gal group and the D-gal + HFD group: injected subcutaneously with D-gal (500 mg/kg/d) in the first 8 weeks, the control group and the HFD group: injected subcutaneously with an equal volume of the vehicle (0.9% saline).	Obesity/Dyslipidemia	The body weights, the levels of plasma TG and TC in the HFD and D-gal + HFD groups were all significantly higher than that in the control group (*P* < 0.001).	The ABR thresholds in the D-gal and D-gal + HFD groups were significantly higher than that in the control group (*P* < 0.01) after 12 months. The ABR thresholds of the d-gal + HFD group were significantly higher at 16 (*P* < 0.05) and 32 (*P* < 0.01) kHz than that in the D-gal group after 12 months.	An increase of abnormal mitochondria, apoptosis and accumulation of mtDNA were shown in the D-gal + HFD group compared to the D-gal group.
C57BL/6J mice, male, 7 weeks old, *N* = 55 (Lee et al., 2020)	Measured body weight weekly, measured glucose tolerance, blood glucose levels and ABR at 12 and 16 weeks, measured TC, HDL-C, LDL-C, TG at 17 weeks.	The diet-induced obesity group: *N* = 45, fed a 60 kcal% fat diet with 30% fructose; the control group: *N* = 10, fed a normal diet; two groups were both fed for 12 weeks from 8 weeks of age	Dyslipidemia/Obesity/Diabetes	The body weight (*P* < 0.01), TC level (*P* < 0.001), LDL-C level (*P* < 0.001), blood glucose level (*P* < 0.001) in the diet-induced obesity group were all significant higher compared with the control group.	The ABR threshold shifts at 16 and 32 kHz were significantly worse in the diet-induced obesity group compared to the control group (*P* < 0.001).	The loss of OHC was significant in the diet-induced obesity group at the basal turn of the cochlea (*P* < 0.05). The diet-induced obesity group showed more apoptosis in the cochlea than the control group (*P* < 0.001).
CD/1 mice, *N* = 60, male, 4 weeks old (Hwang et al., 2013)	Body weight, plasma biochemistry, and ABR were measured at 4 weeks old and 20 weeks old; Omental fat weight measurement and histology of the cochlea were made at 20 weeks old	The diet-induced obesity group: *N* = 30, fed a high fat diet for 16 weeks; the control group: *N* = 30, fed a standard diet of 13.43% kcal fat for 16 weeks	Obesity	Body weight (*P* = 0.0002) and omental fat weight (*P* = 0.0106) were significantly higher in the diet-induced obesity group than that in the control group.	The ABR threshold was significantly higher in the diet-induced obesity group than that in the control group at 32 kHz (*P* = 0.0080) and 16kHz (*P* = 0.0240) sound stimulation.	The mean internal diameter of vessels in the stria vascular was significantly smaller in the diet-induced obesity group at the middle turn (*P* < 0.0001) and the basal turn (*P* < 0.0001). The density of SGNs was significantly lower in the diet-induced obesity group (*P* = 0.0092).
The spontaneously hypertensive rats and the age-paired normotensive Wistar Kyoto rats of both sexes (Tachibana et al., 1984)	Measured the systolic blood pressure, tested the electrocochleography and observed the cochlea at the age of 3, 6, 12, and 20 (for Wistar Kyoto rats only) months	The spontaneously hypertension group and the normotension group	Hypertension	NA	The maximal amplitude of action potentials of the spontaneously hypertensive rats decreased faster with age. The action potentials threshold of the spontaneously hypertensive rats increased faster with age than that of the normotensive rats.	It was showed that the stria vascular was the primary site of cochlear changes in the spontaneously hypertensive rat, followed by the organ of Corti.

ABR, auditory brainstem response; DPOAE, distortion product otoacoustic emission; TC, total-cholesterol; LDL-C, LDL-cholesterol; HDL-C, HDL-cholesterol; TG, triglyceride; NA, not available.

### Diabetes is a high-risk factor for age-related hearing loss (ARHL)

Diabetes is a major chronic disease with an increasing prevalence worldwide. It is estimated that the number of people with type 2 diabetes (T2DM) in the world will reach 700 million by 2045, accounting for 10.9% of the world’s population (Orazumbekova et al., 2022). Diabetes, which is one of the main components of MetS, is also a high-risk factor for ARHL (Mitchell et al., 2009; Helzner and Contrera, 2016; Ren et al., 2017; Mujica-Mota et al., 2018; Kim et al., 2020a). Vasilyeva et al. (2009) induced type 1 diabetes (T1DM) and T2DM in CBA/CaJ mice and found that the ABR threshold increased in each group, in line with the characteristic performance of ARHL. Two months following the induction of diabetes, both the T1DM group and the T2DM group exhibited a significant decrease in distortion product otoacoustic emission amplitude at higher frequencies, while the control group showed no significant change. This suggests that the induction of diabetes in adult CBA/CaJ mice may exacerbate ARHL.

### Dyslipidemia may lead to age-related hearing loss (ARHL)

With an increasing abundance of food and changes in eating habits, an increasing number of people worldwide suffer from dyslipidemia. In the United States, approximately 31 million adults have total cholesterol levels > 240 mg/dL (Karr, 2017). MetS-related dyslipidemia mainly presents as elevated triglyceride (TG) levels and decreased high-density lipoprotein cholesterol (HDL-C) levels, both of which contribute to ARHL (Simpson et al., 2013; Tang et al., 2019). Hyperlipidemia usually leads to atherosclerosis, which manifests as spiral modiolar artery (SMA) stenosis and endothelial dysfunction in the cochlea. Atherosclerosis may impair the cochlea blood supply and lead to ischemia and hypoxia damage. As a result, hair cell (HC) loss (Guo et al., 2005; Lee et al., 2020) and spiral ganglion neuron (SGN) damage are aggravated (Kim et al., 2020b), and the progression of ARHL is therefore accelerated.

With three isoforms (ApoE-ε2, ApoE-ε3, and ApoE-ε4), ApoE performs the function of distributing/redistributing lipids in various tissues and cells of the body and plays a central role in lipid metabolism (Huang and Mahley, 2014). A study showed that the presence of the APOE-ε4 allele was associated with ARHL and presented a dose-response effect (Kurniawan et al., 2012). When wild-type (WT) mice and ApoE knockout (KO) mice with a C57BL/6J background were fed a normal diet for 16 weeks, it was found that the blood lipids and auditory brainstem response (ABR) thresholds of the aged ApoE KO mice were higher compare to the WT group. When ApoE KO mice were fed a high-fat diet, their ABR thresholds increased more significantly (Kim et al., 2020b).

Sirt3 is localized in mitochondria and is involved in lipid metabolism (Anamika et al., 2019). It may promote triacylglycerol use and fatty-acid oxidation by deacetylating and activating long-chain acyl-CoA dehydrogenase (LCAD) (Santos et al., 2021). Sirt3-KO mice on a high-fat diet showed accelerated progression of MetS, including hyperlipidemia (van de Ven et al., 2017). In ARHL, Sirt3 expression was significantly reduced in HCs and SGNs (Takumida et al., 2016). After 10 months of calorie restrictions (CR) in both WT and Sirt3-KO mice (C57/BL6J background), the Sirt3 level in the cochleae of WT mice increased, and the progression of ARHL was delayed, while ARHL in Sirt3-KO mice showed no amelioration (Han and Someya, 2013). Mice with hyperlipidemia and ARHL showed more severe damage of HCs and SGNs and hearing loss compared to mice with ARHL only (Miwa, 2021).

### Obesity had negative effects on age-related hearing loss (ARHL)

Obesity, especially abdominal obesity, is an important diagnostic condition in MetS. Nowadays, the prevalence of obesity has reached 711.4 million worldwide, including 603.7 million adults and 107.7 million children (Afshin et al., 2017). Obesity itself and its comorbidities have crucial effects on hearing loss (Dhanda and Taheri, 2017). As an important indicator of obesity, body mass index (BMI), is a risk factor for ARHL (Han et al., 2018). In a cross-sectional study of elderly people (average age, 66.1 years) in Rotterdam designed by Croll et al. (2019), researchers found that, after adjusting for other influencing factors, subjects with higher BMIs had more severe hearing loss.

Adiponectin (APN), an adipocytokine released from adipose tissue, negatively correlates with BMI (Parida et al., 2019). The levels of APN in women are higher than those in men (Ohman-Hanson et al., 2016). APN increases glucose and fatty acid metabolism by activating adenosine monophosphate-activated protein kinase (AMPK), leading to an increase in insulin sensitivity (Atawia et al., 2019). The reduction of APN plays a key role in obesity (Achari and Jain, 2017). According to a cross-sectional study in Taiwan (Hwang et al., 2011), there is a negative association between APN levels and hearing thresholds, especially at high frequencies. Altered plasma APN levels may contribute to obesity-associated ARHI (Hwang et al., 2011). APN was seen as a significant and independent predictor of ARHL (Tanigawa et al., 2014).

### Hypertension aggravates the development of age-related hearing loss (ARHL)

Hypertension is one of the main chronic diseases whose prevalence is increasing yearly, and its prevalence has a strong relationship with age. Many studies have shown that individuals 55–65 years of age have a > 90% risk of hypertension (Benetos et al., 2019). Hypertension is not only a common disease in elderly individuals but also a component of MetS and has shown a significant relationship with the incidence and the progression of ARHL (Rolim et al., 2018; Kim et al., 2020a). In a study reported by Przewozny et al. (2016), 32 patients with arterial hypertension (≥ 140/90 mmHg) aged 53.1 ± 10.3 years and 32 healthy volunteers matched for age and sex were selected. They conducted expanded tonal audiometry and distortion product otoacoustic emission testing to evaluate the peripheral auditory system, and the minimum auditory angle test at the binaural level for eight azimuths with binaural stimulation was used to evaluate the central auditory system. The results showed that, compared to the control group, the hypertension group not only had poor peripheral hearing (mainly at higher frequencies), but their capacity for sound localization was also significantly reduced.

### The negative influence of metabolic syndrome (MetS) on hearing relates to the number of diagnostic components met

Rim et al. (2021) designed a study to explore the relationship between MetS and sensorineural hearing loss in Korea. The results showed that MetS subjects had higher mean pure-tone hearing thresholds than subjects without MetS in all age groups (*P* < 0.001); moreover, among MetS subjects, there was a significant association between the number of MetS diagnostic components met and the hearing loss rate (*P* < 0.0001). After adjustment for the effects of age and gender, each diagnostic component (e.g., waist circumference, blood pressure, triglycerides, and hyperglycemia) were strongly associated with hearing loss (*P* < 0.001). The results showed that MetS is associated with sensorineural hearing loss, and the number of diagnostic components met was positively correlated with the incidence of hearing loss. A cross-sectional study on middle-age and older Chinese population (mean age, 67.5 years) has also shown a significant association between MetS/its components (including central obesity, low HDL-C levels and hyperglycemia) and hearing loss (Han et al., 2018). Considering age of the participants, the results may serve as an evidence for the relationship between MetS/its components and ARHL.

In addition, drinking, smoking, and a high-fat diet were risk factors not only for MetS (Cheng et al., 2019) but also for hearing loss. Various studies have shown that a high-fat diet and alcohol abuse are associated with hearing loss (Rosenhall et al., 1993; Momi et al., 2015). Also, both active and passive smoking are associated with hearing loss and presented a dose-response effect (Dawes et al., 2014), and smoking and diabetes have a synergistic impact on ARHL (Bae et al., 2020). Therefore, MetS is not only related to ARHL itself; its risk factors are also harmful to hearing, which further implies a connection between MetS and ARHL.

## The pathways of metabolic syndrome (MetS) leading to age-related hearing loss (ARHL)

### The mitochondrial mechanisms of diabetes-induced age-related hearing loss (ARHL)

A recent study showed that elevated blood glucose levels can cause oxidative stress and mitochondrial dysfunction, leading to SGN damage and HC apoptosis (Lyu et al., 2021). In the cochleae of diabetic mice, HCs from the basal turn of the cochlea were significantly damaged, and the number of synapses ribbons on the inner HCs and the blood flow of the cochlea were both reduced. It was also found that the mitochondria of SGNs, synapses, HCs, and stria vascularis were swollen and distorted, showing structure and function damage (Lyu et al., 2021).

Mitochondrial DNA (mtDNA) mutations, such as the mt3243 A > G mutation, can lead to mitochondrial diabetes combined with sensorineural hearing loss. Therefore, it is possible that mitochondrial function may play a vital role in the regulation of blood glucose and the development of hearing. Uncoupling protein 2 (UCP2) is an important transport protein encoded in the nucleus and located in the mitochondrial inner membrane. It can uncouple the oxidative phosphorylation of the mitochondrial respiratory chain and reduce the production of ATP. It was reported that the UCP2-866G/A polymorphism had a significant promotional effect on diabetes in a Chinese population (Hou et al., 2020). It can promote the expression of UCP2 in pancreatic β-cells, which can reduce ATP production, therefore reducing insulin secretion and increasing the blood glucose level (Li et al., 2019). UCP2-866G/A polymorphism was also reported as a high-risk factor for ARHL in India (Manche et al., 2016). Therefore, the high expression of UCP2 caused by UCP2-866G/A polymorphism is a high-risk factor not only for hyperglycemia but also for ARHL.

Sirt1 is a deacetylase expressed on cell nuclei. Down-regulation of Sirt1 expression can regulate the activation of PGC-1α by deacetylation, leading to reduced mitochondrial biogenesis. Research has observed mitochondrial dysfunction and the promotion of apoptosis because of this phenomenon (Tang, 2016). Sirt1 can promote hepatic gluconeogenesis and reduce the blood glucose level by regulating the deacetylation of PGC-1α (Cao et al., 2016), and the reduction of Sirt1 expression may also lead to insulin resistance in the liver and muscle through mitochondrial dysfunction (Cao et al., 2016; Zhou et al., 2018). Supplementation with resveratrol, a kind of Sirt1 agonist, can increase the activation of PGC-1α, which can promote mitochondrial biogenesis, increase the number of mitochondria, and improve mitochondrial function, thereby improving insulin sensitivity in muscle cells of diabetic mice (Zhou et al., 2018). In an ARHL model, the expression of the Sirt1/PGC-1α signaling pathway in HCs was reduced (Takumida et al., 2016; Hao et al., 2019; Pardo and Boriek, 2020) and mitochondrial dysfunction appeared, leading to a severe loss of HCs (Hao et al., 2019). After resveratrol treatment in aged C57B/L mice, there was a significant reduction in age-related auditory threshold shifts and alleviation of outer hair cell (OHC) damage in the apex (Pang et al., 2019). What’s more, further down-regulation of the expression of Sirt1/PGC-1α signaling could further raise the incidence of ARHL by increasing HCs apoptosis (Hao et al., 2019). Therefore, reduced Sirt1 levels and a consequential decrease in mitochondrial biogenesis are closely correlated to the occurrence of both diabetes and ARHL.

### The mitochondrial mechanism of dyslipidemia leading to age-related hearing loss (ARHL)

According to studies, atherosclerosis caused by hyperlipidemia can cause ischemia–hypoxic damage in the cochlea, leading to mitochondrial dysfunction, which consequently decreased ATP production and increased ROS levels. In addition, HC apoptosis increased as a result (Guo et al., 2005; Lee et al., 2020). In mice with hyperlipidemia and ARHL, an increase in the accumulation of ROS in SGNs was found, leading to more severe SGN apoptosis (Kim et al., 2020b).

ApoE-ε4 not only plays an important role in lipid metabolism; its fragments can also target the mitochondria of neurons, causing neurotoxicity and mitochondrial dysfunction (Huang and Mahley, 2014). Compared to non-carriers, ApoE-ε4 carriers had significantly lower expression of mitochondrial antioxidant enzymes (Yin et al., 2020), which could lead to the abnormal accumulation of ROS and more severe oxidative stress (Smith et al., 1998). Compared to aged C57BL/6J mice on a normal diet, the increase in ABR thresholds in aged ApoE KO mice on a normal diet was due to the rise in ROS accumulation in SGNs and the consequent increase in SGN damage. All these changes were more significant in aged ApoE KO mice on a high-fat diet (Kim et al., 2020b). The above-mentioned studies showed that ApoE not only affected lipid metabolism but also affected ARHL by increasing the accumulation of ROS and the apoptosis of SGNs. ApoE isoforms are important factors that affect both hyperlipidemia and ARHL.

Atherosclerosis caused by hyperlipidemia is an important pathological change resulting in cochlear dysfunction and may correlate with an imbalance in ROS homeostasis and mitochondrial dysfunction (Di Lisa et al., 2009). Sirt3 is localized in mitochondria (Anamika et al., 2019) and plays an important role in maintaining mitochondrial function. It can activate antioxidant enzymes, such as isocitrate dehydrogenase (IDH2) and superoxide dismutase 2 (SOD2) by deacetylation so that the accumulation of ROS and the oxidative stress damage in cells can be reduced (Chen and Tang, 2014; Singh et al., 2018). Increasing the expression of Sirt3 could prevent many age-related diseases (van de Ven et al., 2017). In an ARHL mouse model, the expression of Sirt3 was significantly reduced in HCs and SGNs (Takumida et al., 2016). Calorie restriction can up-regulate the expression of Sirt3 in WT mice, thereby increasing the activation of IDH2, triggering a delay in the progression of ARHL. Meanwhile, in Sirt3-KO mice, the ABR thresholds showed no improvement after calorie restriction (Han and Someya, 2013). A significant decrease in Sirt3 expression was observed in mice with both hyperlipidemia and ARHL (Miwa, 2021). This suggests that decreased expression of Sirt3 can inhibit deacetylation, which is a common risk factor for both hyperlipidemia and ARHL.

Since mitochondria are the site of β-oxidation of fatty acid, the normal function of mitochondria is an important factor in lipid metabolism. DRP-1 is one of the main regulators of mitochondrial fission, where it regulates the balance of mitochondrial fission/fusion and can thereby affect mitophagy, and it is essential for the maintenance of normal morphology and the function of mitochondria (Ma et al., 2020). At the same time, DRP-1 also plays an important role in lipid metabolism. In cardiomyocytes and Hela cells, the increased/decreased expression of DRP-1 can conversely inhibit/enhance the expression of carnitine palmitoyltransferase 1 (CPT1), a rate-limiting enzyme of mitochondrial β-oxidation, and lead to abnormal lipid metabolism (Wang et al., 2020; Song et al., 2021). In the HEI-OC1 cell model and cochlear explants model of ARHL, the expression of DRP-1 was reduced, showing mitochondrial dysfunction and reduced mitophagy level. After C56BL/6J mice were treated with Midiv, a mitochondrial fission inhibitor, it was observed that they showed higher ABR thresholds, lower DRP-1 expression, and down-regulated mitophagy levels when compared to mice with only ARHL (Lin et al., 2019). Interestingly, age can conversely cause an increase in mitophagy in the vasculature of mice, and induction of acute hypercholesterolemia in aged mice can further up-regulate mitophagy levels in the aorta (Tyrrell et al., 2020). Therefore, mitochondrial dynamics and mitophagy levels may vary in different tissues of the body in hyperlipidemia and ARHL. In conclusion, DRP-1 plays an important role in both hyperlipidemia and ARHL by affecting mitochondrial fission and mitophagy.

### Obesity-induced age-related hearing loss (ARHL)driven by mitochondrial mechanism

Now, there are still few studies on the inter-relationship between mitochondrial dysfunction, obesity, and ARHL. According to Tanigawa et al. (2014), when comparing APN-KO and WT mice (C57BL/6 background), it was found that APN-KO mice had earlier onset of ARHL, and decreased blood flow in the stria vascularis, and greater loss of SGNs and HCs. Supplementation with exogenous APN can ameliorate ARHL, although the mechanism at play is still unknown (but may be related to mitochondrial dysfunction). Studies showed that increasing the concentration of APN in aged Sprague–Dawley rats increased the activation of the AMPK/PGC-1α signaling pathway. Consequently, there were an increase in mitochondrial biogenesis and an amelioration of mitochondrial dysfunction in skeletal muscle (Liu et al., 2019). Therefore, we guess that a reduced adiponectin level may lead to a decrease in the activation of AMPK and affect mitochondrial biogenesis, having a promotive effect on obesity and ARHL.

In addition, how obesity affects hearing may also be related to insulin-like growth factor 1 (IGF-1). IGF-1 is crucial in the regulation of energy metabolism, while energy is mainly provided by mitochondria in the form of ATP (Schell et al., 2021). In humans, IGF-1 levels not only decreased with increasing BMI (Fowke et al., 2010; Crowe et al., 2011) but also decreased with age, and the rate of descent with age is higher in men than women (Kucera et al., 2015). In the cochlea, IGF-1 is highly expressed in SGNs and stria vascularis. The concentrations of IGF-1 decrease with age in the cochlea (Riva et al., 2007; Riquelme et al., 2010), which causes more severe degeneration of SGNs, leading to ARHL (Riquelme et al., 2010). Intratympanic injection of IGF-1 can effectively control or even reverse sensorineural hearing loss (Dave et al., 2021). When the HCs of zebrafish were treated with neomycin and thus exposed to oxidative stress, increasing the bioavailability of IGF-1 could promote the maintenance of normal mitochondrial function, reduce the production of ROS, and therefore decrease HC loss (Alassaf et al., 2019). Therefore, decreased expression of IGF-1 may not only contribute to obesity, but also have an important impact on ARHL by affecting mitochondrial function.

### The mechanism of hypertension leading to age-related hearing loss (ARHL): From a mitochondrial dysfunction perspective

Hypertension is often accompanied by the spasm of arterioles and remodeling of blood vessels. The cochlea is also one of the target organs of hypertension. During hypertension, increased secretion of angiotensin II (Ang-II) not only leads to vasoconstriction but also increases mitochondrial oxidative stress levels by reducing nitric oxide bioavailability and down-regulating PGC-1α (Forte et al., 2020). Hypertension mainly affects the stria vascularis (Tachibana et al., 1984), which nourishes the organ of Corti in the cochlea, leading to vascular damage, endothelial dysfunction, and thickening of stria vascularis such that the partial pressure of oxygen in the cochlea was decreased and the balance of potassium ion circulation was disrupted, causing HC loss and hearing loss (Przewoźny et al., 2015).

**FIGURE 2:**
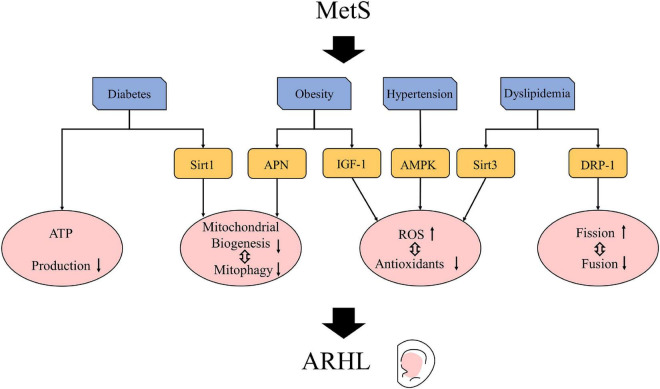
Possible mechanisms by which metabolic syndrome (MetS) affects age-related hearing loss (ARHL). Mets may cause signal pathway and related factor changes, such as insulin-like growth factor 1 (IGF-1) (Riquelme et al., 2010; Alassaf et al., 2019), adenosine monophosphate-activated protein kinase (AMPK) (Zhao et al., 2020), sirtuins (Han and Someya, 2013; Hao et al., 2019; Pang et al., 2019; Miwa, 2021), adiponectin (APN) (Liu et al., 2019) and DRP-1 (Lin et al., 2019). These changes lead to mitochondrial changes so that can exacerbate ARHL.

Hypoxic vasoconstriction caused by hypertension was one of the main factors affecting the cochlear function, and the activation of AMPK was one of its important mechanisms (Zhao et al., 2021). As an important regulator of the energy homeostasis of cells, AMPK is often activated when there is oxidative stress or the ratio of AMP/ATP increases. During pulmonary hypertension, physiological hypoxia causes mitochondrial dysfunction in pulmonary vascular smooth muscle cells, reducing ATP production and increasing ROS production; thus, the activation of AMPK increases, leading to vasoconstriction (Zhao et al., 2021). The Tg-mtTFB1 (Tg-B1) mouse with a C57/BL6J background is a mouse model of mitochondrial deafness that manifests a mitochondrial homeostasis imbalance and a consequent increase in ROS production. In Tg-B1 mice, the ROS-dependent activation of AMPK is increased, which can increase OHC and SGN apoptosis through the signaling pathway of ROS-AMPK-E2F2, leading to late-onset, progressive hearing loss. In aged Tg-B1 mice, the ABR thresholds were significantly higher than that in WT mice (McKay et al., 2015). Meanwhile, the AMPK^±^ Tg-B1 mice in whom the expression of AMPK was reduced had similar hearing levels as the WT mice (AMPK^+/+^). The AMPK^±^ Tg-B1 mice showed a reduced loss of OHCs and SGNs and a significantly improved function of stria vascularis compared to the Tg-B1 mice (McKay et al., 2015; Zhao et al., 2020). These findings indicate that the excessive activation of the AMPK pathway caused by an increase in ROS plays an important role in ARHL. Reducing the expression of AMPK can rescue hearing loss by reducing ROS. In short, mitochondrial dysfunction can lead to vasoconstriction and hearing loss through the ROS/AMPK pathway, which may be one of the common pathogenic mechanisms of hypertension and ARHL (Figure 2).

## Potential treatment and prevention: Target for mitochondrial dysfunction

As mentioned above, mitochondrial dysfunction may be one of the common mechanisms to both MetS and ARHL, so improving mitochondrial function can be considered as a potential target for the treatment and prevention of these two diseases.

As one of the easiest interventions to implement, life interventions, such as physical exercise and CR, are always considered as the first and most important prevention/treatment. Many studies have shown that both physical exercise and CR have positive effects on MetS and mitochondrial function (Menshikova et al., 2017; Kunduraci and Ozbek, 2020). Mitochondrial dysfunction is relevant to the development of insulin resistance, obesity, and type 2 diabetes (Lahera et al., 2017; Pinti et al., 2019). Physical exercise can activate PGC-1α, leading to promotion of mitochondrial biogenesis (Bhatti et al., 2017). CR can reduce the accumulation of ROS and increase the expression of antioxidant enzymes (Waldman et al., 2018) so that the oxidative stress can be reduced. Previous studies have shown that reduced physical activity is one of the main related factors for many metabolic diseases (Bhatti et al., 2017), CR can elevate the insulin sensitivity (Bhatti et al., 2017), CR interventions combined with resistance training can significantly reduce the presence of MetS in overweight/obese older adults (Normandin et al., 2017). In the study designed by Mthembu et al. (2022), 17 overweight/obese older adults were assigned to CR intervention or physical exercise intervention (5 days/week, moderate-intensity) for 16weeks. It was found that CR significantly increased citrate synthase activity (*P* ≤ 0.04) while exercise increased mitochondrial volume density (*P* < 0.05), both interventions had similar improvements in insulin sensitivity (*P* < 0.04). Down-regulated mitochondrial biogenesis and reduced antioxidant enzymes can also be found in ARHL mice (Chen et al., 2017; Oh et al., 2020). CR has been reported to delay the progression of ARHL through increasing the activation of IDH2 in aged C57BL/6J mice (Han and Someya, 2013). Long-term physical exercise had also been reported that can delay the progression of ARHL in CBA/CaJ mice (Han et al., 2016). Therefore, life interventions (physical exercise and CR) are a viable option to prevent/treat ARHL and MetS.

Several nutritional interventions and pharmacological interventions have been proved to prevent the development of MetS and ARHL by improving mitochondrial dysfunction. Supplementation of resveratrol can activate Sirt1, leading to activation of PGC-1α. This activation event has been proved to promote mitochondrial biogenesis and mitophagy, therefore improving the balance of mitochondrial turnover (Tang, 2016). Previous studies reported an improvement of ARHL in aged mice with supplementation of resveratrol and an attenuation of insulin resistance in skeletal muscle cells with overexpression of Sirt1, both of which have been shown to be related to mitochondrial dysfunction (Zhang et al., 2015; Hao et al., 2019). To reduce the oxidative stress, some antioxidants, such as alpha-lipoic acid and mitoquinone (MitoQ), can also be considered as potential nutritional interventions. Alpha-lipoic can ameliorate the components of MetS and slow down the onset of MetS (Pershadsingh, 2007). A previous study indicated that supplementation of alpha-lipoic acid improved mitochondrial function in the cochlea of aged Fischer rats and delayed the development of ARHL (Seidman et al., 2000). MitoQ, a mitochondria-targeted antioxidant, has been shown to not only reduce ROS production, but also to improve the balance of mitochondrial turnover and mitochondrial dynamics, leading to better mitochondrial function and health (Marín-Royo et al., 2019; Yang et al., 2021). Recently, the attenuation of MetS and its components in human and experimental animals have been reported (Zinovkin and Zamyatnin, 2019), in which these improvements were considered to play an ameliorative role (Yang et al., 2021). MitoQ has been reported to reduce ROS-induced mitochondrial damage and therefore protect HCs from apoptosis in IDH2-deficient mice (Kim et al., 2019). Kim et al. speculated that MitoQ may be a potential therapeutic target for ROS-induced hearing loss because of that. There are no studies on the effect of MitoQ on ARHL yet. Since increased oxidative stress can be found in both ROS-induced hearing loss and ARHL, there is a possibility that MitoQ may be a potential therapeutic target not only for ROS-induced hearing loss, but also for ARHL.

## Perspective

After reviewing all the aforementioned reports, we found that both MetS and its risk factors had varying degrees of influence on ARHL. The more diagnostic components that were matched, the higher the rate of hearing loss was. In terms of the mechanism, mitochondrial function changes in cells played an important role. In ARHL, age affects hearing mainly by causing an imbalance in mitochondrial redox homeostasis, mitochondrial turnover, and mitochondrial fission/fusion, thereby affecting mitochondrial morphology and function. For MetS and its diagnostic components, its progress is related to IGF-1, AMPK, sirtuins, and other signaling pathways, which may also play an important role in ARHL by mitochondrial dysfunction. Therefore, we guess that mitochondrial dysfunction may be one of the common pathogenic mechanisms of MetS and ARHL. Through these signaling pathways, MetS may aggravate mitochondrial dysfunction, consequently aggravating ARHL. This proposes some possible therapeutic targets for the treatment and prevention of MetS combined with ARHL, and it is also helpful for its clinical diagnosis, but the mechanism remains to be further studied.

In addition, in the cochlea, studies are needed regarding the changes in these signaling pathways in MetS and ARHL; the expression changes of these pathways may differ based on the different diagnostic components of MetS. Therefore, more research is needed to find out how these signaling pathways change and interact when ARHL is combined with MetS. At present, studies on the relationship between MetS and ARHL are still focused on epidemiology, and most of them are cross-sectional studies. At the same time, there is no grade classification for the influence of MetS on hearing according to the number and types of components of MetS, nor are there diagnostic or treatment guidelines for each grade. As such, additional research on the influence of MetS on ARHL from the perspective of mitochondrial dysfunction could help us to better understand the mechanism so that we can better explore the treatment and prevention of ARHL to better protect auditory function from damage.

## Author contributions

MX made substantial contributions to the design of the work and approved the final version of this article. DG, BY, AZ, TZ, RD, KC, YP, and PJ participated in the conception and design of the study, collected the literature and wrote the manuscript. All authors agreed to be accountable for all aspects of the work in ensuring that questions related to the accuracy or integrity of any part of the work are appropriately investigated and resolved. All authors contributed to the article and approved the submitted version.

## Footnotes

1
http://www.stats.gov.cn/tjsj/pcsj/
